# Key issues of health and safety for workers in residential aged care: An expert study

**DOI:** 10.3389/fpubh.2022.1041949

**Published:** 2023-01-06

**Authors:** Liz Seaward, Damian Morgan, Alana Thomson

**Affiliations:** ^1^Victorian Institute of Health and Safety, Institute of Health and Wellbeing, Federation University Australia, Ballarat, VIC, Australia; ^2^College of Business, Law and Governance, James Cook University, Townsville, QLD, Australia; ^3^Institute of Health and Wellbeing, Federation University Australia, Brisbane, QLD, Australia

**Keywords:** aged care OHS, worker injury, residential aged care worker injury, worker health and safety, occupational health and safety, aged care sector, demographics and OHS, physical and emotional work

## Abstract

**Introduction:**

Residential aged care (RAC) represents a fast-growing sector within Australia's health care system and is characterized by high levels of workplace injury. To better understand this injury problem, this study investigated key informant perspectives concerning sector occupational health and safety (OHS) focused on key issues associated with the risk of worker injury.

**Method:**

Semi-structured interviews were undertaken with nine key informants representing (OHS) specialists, healthcare employers, regulators, worker association representatives, and academic researchers in OHS or healthcare. Interviews were transcribed verbatim and analyzed using thematic analysis.

**Results:**

This study identified six themes on OHS within RAC including (i) the physical and emotional nature of the work, (ii) casualization of employment, (iii) prioritization, (iv) workforce profile, (v) OHS role construction, and (vi) clinical standards. The study highlighted differences in OHS roles between RAC and other safety-critical sectors regarding governance and management of OHS. The key informants identified a propensity within RAC to downplay or disregard worker OHS issues justified through prioritizing resident safety. Further, neither OHS professional nor institutional logics are prominent in RAC leadership and decision-making where the emphasis is placed on mandatory standards to maintain funding purposes. Several recommendations are made to address identified issues.

## 1. Introduction

The aged care sector represents one of the largest employer groups in Australia (1). The sector continues to experience growth in supporting the needs of the country's aging population (2). Sector employees provide direct resident care or hold positions in cleaning, catering, laundry, and other services. Australia's peak organization responsible for worker health and safety, Safe Work Australia, regards direct health care workers as “a key risk group due to the very nature of the work they do on a daily basis” [(3), para 3].

A subset of the aged care sector is residential aged care (RAC). RAC service providers deliver 24-h care outside the home to persons requiring significant assistance (4), including for activities of daily life such as bathing, eating, and moving about (5). RAC work is both physically and psychologically demanding (6–8). Physical demands are associated with activities of daily resident life such as bathing, dressing, and moving from beds. Psychosocial demands stem from high workloads, low job control, resident verbal aggression, and emotional aspects of the job (6). Commonly reported injuries among RAC workers include sprains/strains and chronic joint or muscle conditions, as well as stress and other psychosocial conditions (9). Significant evidence shows too that psychosocial hazards impact workers physically by increasing the risk of musculoskeletal disorders (10). The hazardous nature of RAC work is acknowledged by the sector regulator and researchers. As an example, one source of injury data found that 14% of 8,885 direct care workers surveyed reported a work-related injury or illness during 2016–2017 (9).

Despite the physicality of RAC work, aged care work has been labeled “women's work” [(11), p. 112] due to its similarity with unpaid care work that is traditionally carried out by women. Aged care work in Australia is particularly gendered with female workers comprising 87% of RAC workers, and 32% of aged care workers are born overseas (9). Female, migrant, and/or culturally and linguistically diverse (CALD) workers are often considered vulnerable through their employment in casual, short, or fixed-term contracts and agency work (12). Up to 20% of aged care workers fall within these precarious employment arrangements making them vulnerable to limited job security and a lack of leave entitlements (11, 13, 14). Vulnerable and precarious work not only exposes workers to unfavorable work arrangements but also to hazardous conditions (12). Workers with English as a second language also have a higher likelihood of injury resulting from communication difficulties (15) and may be reluctant to identify safety concerns (16). Scarino et al. (15) suggested that this situation arises when questioning supervisors or colleagues is considered culturally disrespectful (15).

RAC services are delivered by not-for-profit, private, and public sector organizations. The sector is regulated and accredited under the Federal Government's Aged Care Act 1997 (Cth) (referred to in the following as “the Act”). The Act includes a prescribed funding framework and requires RAC service providers to be accredited with a set of eight Aged Care Quality Standards (ACQS) for compliance (17). The quality standards each focus on a different aspect of consumer outcomes (17). As an example, one quality standard covers infection control, which became a significant compliance focus during the COVID-19 pandemic. Worker health and safety (OHS) is not a specific quality standard within the funding framework or the accreditation and audit process (4).

The demands of healthcare workers are undertaken typically by personal care assistants (PCAs). There is no specification for PCAs to hold a minimum qualification, nor is there a registration or accreditation process for such roles (4). Hence, PCAs meet no industry-specified standards for OHS literacy or training. The potential result may be OHS shortfalls through, for example, miscommunication or worker reticence in clarifying critical information (4, 16). Consistent with the broader workforce profile, the PCA workforce profile is predominantly women from culturally and linguistically diverse (CALD) backgrounds (2).

**Figure 1:**
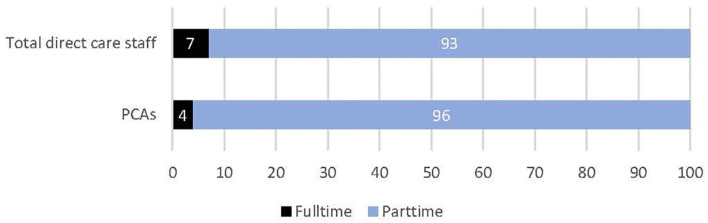
Percentage of permanent part time to full time direct care staff. Data source was sourced from the following publication and formatted into a graph (14).

**Figure 2:**
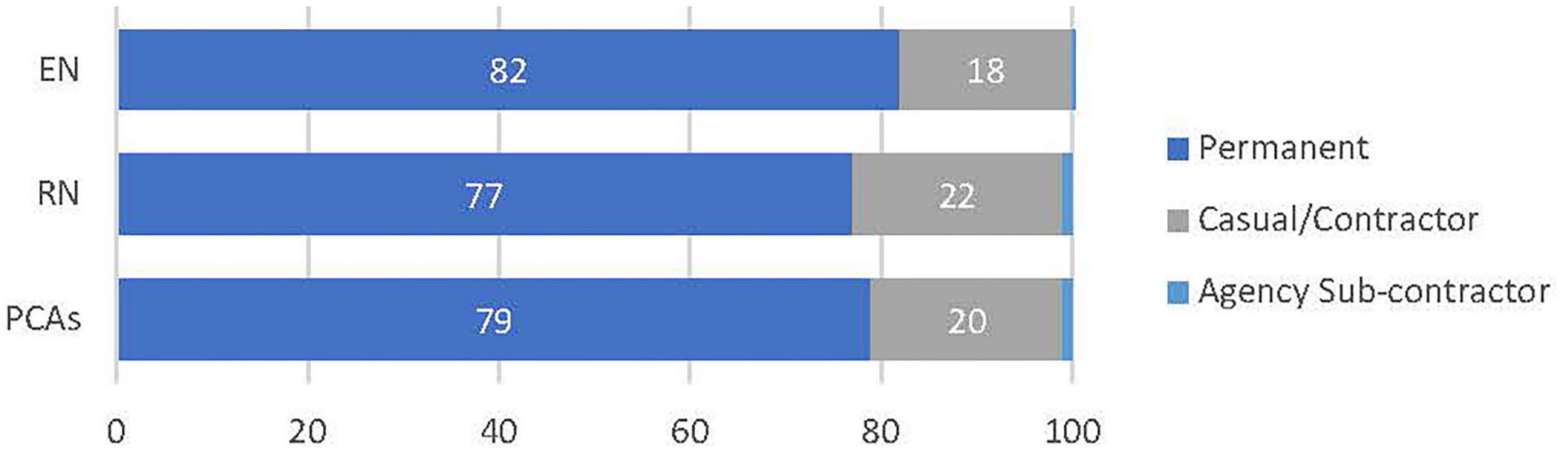
Proportion of direct care workers in each employment type. Data source was sourced from the following publication and formatted into a graph (14).

Figures 1, 2 display relative RAC worker proportions by forms of employment, based on data from the Department of Health (14). Most permanent PCAs are engaged on a part-time basis (Figure 1) (14) while overall, some 21% of PCAs are employed as casuals or on contract (Figure 2) (14). This infrequent work-engagement profile is compounded by a significantly under-resourced workforce (2).

Recently in Australia, nationwide concerns for persons in resident care prompted a Royal Commission into Aged Care Quality and Safety (2018–2021) (2), which provides evidence that workforce development and OHS exhibit a leadership deficit and have been underfunded and undervalued. The Royal Commission concluded that many of these issues existed pre-COVID, however, COVID likely exacerbated these challenges. Despite these long-term issues, there have been few investigations of key issues for the health and safety of workers in RAC in Australia. This is surprising given the high frequency of RAC work-related injuries relative to other sectors (4, 18). Further research into the key issues for the health and safety of workers is justified due to the projected growth of the sector and the complexity of the issue. First, the number of RAC workers is expected to grow significantly in response to forecasted increases in the aging population and the corresponding demand for supporting services (6). Higher demand will likely result in increased worker injuries given that risk factors are not well understood (19). Second, OHS issues in healthcare are considered wicked problems (20–22). Goh et al. describe wicked problems as “messy” [(23), p. 118] since they cannot be easily defined, and proposed solutions may make existing problems worse. Considering the sector and OHS from a wicked problem perspective, and seeking to understand the complexity of the challenges, may prove a useful approach. To date, much research has focused on individual factors and ignored the complex nature of many RAC worker injuries (6). For instance, the complex nature of RAC OHS and RAC injuries, which involve both physical and psychosocial contributors, suggest simple countermeasures may not be effective in creating safer RAC work environments.

To address this gap, the study reported here questioned key informants about issues pertaining to RAC worker OHS. These issues concerned both general insights on OHS practice and issues arising from the COVID-19 pandemic onset. Specifically, the study aimed to better understand key issues associated with RAC OHS and to identify priority areas for future research. Before describing the method and results, a review of extant literature situates the study. Key themes are reported and discussed, and a concluding discussion follows including requirements for further research.

## 2. Literature review

The RAC sector represents a complex environment supported by multiple systems relying upon a diverse workforce undertaking physically and emotionally demanding work (24). Regarding extant literature, two fields of research informed the study: (i) Regulatory systems and OHS management and (ii) OHS professionals and institutional logic.

### 2.1. Regulatory systems and OHS management

Traditionally, the RAC sector has not been deemed a safety-critical sector when compared to aviation, oil and gas production, or nuclear power generation (25). Long-established safety-critical industries are required to implement safety management systems to comply with external regulations (26, 27). Recent literature argues RAC should also be considered safety-critical (25). However, the external regulator for RAC service providers is focused on meeting ACQS accreditation (17) for resident care and service provision. There is no quality standard within the external regulation for RAC that mandates the implementation of OHS management systems (28).

The RAC sector must, like all other Australian employers, meet basic OHS legislation (29). However, OHS legislative compliance does not link to funding or involve mandated audits. Hence, RAC service providers instead prioritize compliance with required quality standards to maintain funding through securing accreditation (28). Typically, frameworks for external accreditation influence an organization's operations (30, 31). For RAC, quality standards compliance demands resourcing and time commitments directed toward accreditation-related activities including preparation for external audits and appraisals (32). As Pomey et al. (33) highlight, maintaining accreditation becomes the RAC's primary concern as this is linked to government funding.

Grote identifies specific considerations for designing effective OHS management systems (34). First, safety management at the organizational level should comprehensively address the specificities of external regulation frameworks and respond to the sector or organizational nuances. However, the gap that exists for RAC is that the ACQS does not include the health and safety of workers (17). Second, Grote highlights the need for the management of safety which aligns with safety processes and personal safety. Grote notes that when process safety and personal safety are disconnected, hazards are presented (34). For example, in RAC a personal hazard may arise where a worker slips, trips, or falls while a process hazard may stem from errors in dispensing medication to residents. From a RAC perspective, personal hazards facing a worker do not expose residents to injury risk while process hazards may not directly expose workers. This dichotomy of risk exposure found in RAC contrasts with safety-critical industries where process and personal safety are aligned (34). Notwithstanding, hazards arising from the COVID-19 pandemic have demonstrated that RAC process safety directly impacts RAC workers where infection (managed as a process hazard) spread among both workers and residents (35).

The disconnect between personal and process safety adds complexity, reducing worker OHS (34). On this, Grote argues that maintaining the health and safety of the worker in such situations requires actions by the organization in addition to their primary task of meeting accreditation standards and process requirements (34). For example, resident bed-making standards may require additional actions including manual handling training, overhead tracking, or mechanized beds, to minimize worker-related hazards (e.g., back strain). However, when resident safety and resource/cost savings are overarching priorities, the low priority of OHS may be compounded by the RAC sector lacking OHS professionals at appropriate decision-making levels (36), discussed next.

### 2.2. OHS professionals and institutional logics

There is a paucity of empirical research on the role of OHS professionals within organizations (37, 38). The broader OHS literature postulates that effective relationships relied upon by OHS professionals derive from critical interactions with senior managers (39–41), power relationships (37, 42), and hierarchical authority or positioning (43, 44). While holding positional authority improves an OHS professionals' capacity to influence senior management (45), the positioning and authority of OHS roles within RAC have not been documented, although anecdotally, high-level OHS roles within these organizations appear uncommon. Regarding qualifications, Oakman et al. report that in questioning 10 RAC specialist OHS managers/coordinators, just three had graduate-level OHS qualifications and two had minimal or no qualifications in OHS (36).

Provan identified institutional logic and institutional work as factors influencing the roles of OHS professionals (37). Institutional logic assumes a particular set of “assumptions, values, beliefs, and rules by which individuals… provide meaning to their social reality” [(46) p. 804]. Institutional logic shapes institutional work represented by organizational actions. Institutional work, conducted alongside operational activities, is constructed by a professional's values, rules, and shared beliefs. These factors influence worker behavior toward what is important (47, 48). Institutional logic within healthcare can often cause conflict in meeting the competing goals of medical standards, care requirements, and managerial aspirations (49). An example is the care institutional logic which follows a worker's professional values and beliefs. This form of logic may push RAC workers to consider residents' health and safety before their own (50). As competing institutional logics rely upon differing interpretations of reality, their existence may exacerbate solving complex OHS problems (49).

Rae and Provan (51) posit that OHS professionals' work practices are a form of institutional work. Specific bodies of knowledge and accreditation frameworks have been developed for OHS pertaining to professional roles and problem-solving (52). In the context of RAC, as outlined earlier, there is a propensity to focus on quality standards and compliance. However, there is tension between this compliance approach and more proactive approaches, particularly in terms of determining new, changing, or emerging hazards and risks. There has been limited empirical research that explores this tension.

The literature review highlights issues potentially impacting RAC OHS performance. These include overshadowing OHS by prioritizing quality standards, high proportions of female and CALD workers disinclined to raise safety concerns, vulnerable workers on insecure work arrangements, and tensions in institutional logic provided by prominently positioned OHS professionals. These propositions warrant further investigation to assess their impact on RAC worker OHS. In line with the previously stated aims, the reported study sought key informant viewpoints and opinions on these issues to uncover priorities for further RAC OHS research.

## 3. Method

### 3.1. Study design

The study employed key informant interviews, a suitable technique for investigating undeveloped research areas (53). The creation of a semi-structured interview guide was informed by a literature review identifying factors underlying the RAC sector and refined following discussion between authors (see Table 1). A semi-structured interview guide was preferred allowing key informants to draw deeply on their own perceptions and views of the health and safety of workers in RAC.

**Table 1:** Interview guide questions.

**1**	**What led you to your current position and the connection with aged care?**
2	What works and what doesn't work in aged care worker health and safety?
3	Do you see any structural challenges within the sector?
Prompt	For example, where OHS sits within a RAC organization and its position?
4	Have you identified any difference between the different types of aged care – for example private, public, and not for profit?
5	What can you tell me about leadership within the sector?
6	I would like to ask about your experiences with leadership in the RAC sector and leadership shown day to day?
7	Have you any thoughts or experiences regarding the accreditation standards as they relate to OHS?
8	Any comments regarding the balance or priorities of worker safety and resident safety?

Ethical approval for the study was granted by the Human Research Ethics Committee, Federation University Australia (project number A19-133).

### 3.2. Key informants and recruitment

Key informants were selected to provide a range of sector and stakeholder perspectives on RAC OHS. A purposive sampling approach was implemented (54). Key informant research utilizes participants chosen for their qualifications, knowledge, and/or specific status in relation to the study (53). The key informants for this study were purposively chosen to also draw on diverse stakeholder viewpoints (53). Five key informants (stakeholder) groups were identified:

1. OHS regulators to gather the perspective of the RAC sector and OHS challenges;
2. Worker association providing broader worker perspectives of OHS;
3. Employer associations to gain the perspective of managers within the sector;
4. Academic researchers in OHS or healthcare can convey holistic or specialized perspectives of the sector and its challenges; and,
5. OHS industry association representatives for a specialized perspective.

Key informants were identified through the lead researcher's professional network. No professional relationships existed between the researchers and key informants, though for the lead researcher, brief unrelated interactions had occurred with two key informants. Initially, direct contact for study participation was made through an email invitation to 11 potential key informants. Nine provided consent to participate in the study, two invitations were unanswered. Table 2 provides a summary of the nine key informants for the study, along with sector/stakeholder group representation or association.

**Table 2:** Summary of key informant stakeholder groups and experience.

	**Key Informant Groups**				
**Key Informant**	**OHS Regulators**	**Worker Association**	**Employer Associations**	**Academic Researchers**	**OHS Industry Associations**
1		10 + years			
2				10+ years Health sector quality, safety, and systems improvement	
3				10+ years OHS and human factors	
4	2 + years				
5					5 + years
6				10 + years OHS and management	
7				10 + years Health sciences, injury research & safety culture	
8			5 + years		
9				10 + years Patient safety	

### 3.3. Interview process

All key informants agreed to interviews being audio recorded. Semi-structured interviews were conducted from November 2020 to May 2021 and ranged between 30 and 60 min in duration. Interview questions and topics are listed in Table 1. Due to COVID-19 restrictions, eight interviews were conducted remotely via video and one by telephone. Interviews were transcribed verbatim using the Microsoft Teams technology (55) with manual corrections or additions from recorded notes.

### 3.4. Data analysis

A qualitative thematic data analysis followed Fereday and Muir–Cochrane's hybrid approach for identifying codes and data patterns. The process followed three stages. In stage one, interview transcripts were reviewed against recordings for accuracy. In stage two, data accounting for data repetition was analyzed among key informants and pattern identification (56). A deductive process aligned to interview questions to identify “meaningful units of text” [(56), p. 87] while an inductive process reflected new themes created for data segments outside a deductively derived theme. In stage three, a review of derived themes was conducted to identify any overlap. Themes were considered and refined by all authors at each stage of the analysis and write-up. Six themes emerged for reporting.

### 3.5. Researcher positionality/bias

The positioning of the lead researcher is acknowledged to have informed the research process (57). The lead researcher has extensive professional experience as an OHS consultant in the Australian RAC sector. This positionality reflects a combination of insider and outsider roles informing the present study. Given the dearth of empirical and scholarly work on the focal topic, this positionality is acknowledged as a strength in the current inquiry. The possible introduction of unintentional bias was acknowledged and challenged during data analysis and interpretation through reflective practice among the research team. To counter any possible bias from insider positionality, initial findings and qualitative themes were reviewed with second and third authors by considering direct evidence from interviews. This process of inquiry prioritized the construction of themes with key informants' own words rather than with the lead researcher's (first author) interpretation and the making of meaning.

## 4. Results

Six identified themes were: (i) Physical and emotional work; (ii) Casualization of RAC work; (iii) Prioritization of OHS and resident safety; (iv) Female, CALD, and aging workforce; (v) OHS role construction and importance; and, (vi) Tension between clinical standards and OHS approaches. Table 3 provides a visual theme summary mapped to key informant evidence. Each theme is now described along with direct quotations from key informants to provide examples and supporting evidence.

**Table 3:** Themes discussed across key informant groups.

	**Key informant groups**				
**Themes**	**OHS regulator**	**Worker association**	**Employer associations**	**Academic researchers**	**OHS industry associations**
Theme 1: Physical and emotional work		✓		✓	
Theme 2: Casualisation of RAC work	✓	✓		✓	
Theme 3: Prioritization of OHS and resident safety	✓	✓	✓	✓	
Theme 4: Female, CALD and aging workforce	✓	✓	✓	✓	
Theme 5: OHS role construction and importance	✓	✓		✓	✓
Theme 6: Tension between clinical standards and OHS approaches		✓			✓

### 4.1. Physical and emotional work

Theme 1 provides key informant insights on the health and safety of workers as influenced by high levels of physical work and emotional demands. Key informants noted that the seriousness of OHS risks had only recently been acknowledged by the industry and wider community. Furthermore, limited budgets and available resourcing for RAC facilities were noted as factors exacerbating the nature of RAC work.

The pool of RAC candidate workers is limited, indicating that persons unsuited to RAC positions may take up employment. The following quote from an academic key informant describes the situation where an unprepared worker suffered consequences, ending for a time their RAC employment:

An organization appointing a 50-year-old woman into a key support role. She was relatively unfit. And then… [the RAC work required] a lot of physical, manual…, ongoing lifting and work, day in day out… Within 4 months she is off on stress leave with both physical and also emotional needs.

Key Informant 2

Key informant 1 stated that the regulator has historically paid little attention to the RAC sector. As this key informant explains, RAC is becoming recognized as a safety-critical sector of employment:

It's taken [the OHS regulator] until the last 3 years or so to even recognize health [and the RAC sector] as a hazardous industry… We have had to …push and upskill [the regulator] to understand what is going on in health.

Key Informant 1

Competing demands of worker OHS contextualized by limited budgets and resources were reported by several key informants. Key informant 3 described the RAC sector as “incredibly understaffed”. The consequential result is underserviced clients due to RAC workers' inability to perform important resident care roles. This is also reflected in the following comment related to the effects of funding constraints:

I think the general outcomes are showing that… [where profit is] one of the goals… [for-profit providers] have to cut services. The biggest cost is staffing costs. You can have one less TV in the room or …. you can cut down staff, which is one of the longer-term challenges.

Key Informant 2

Limited resources and staff cutbacks compound risks posed due to the physical and emotional nature of RAC work. The limited support provided to available staff for addressing and minimizing OHS risks is highlighted in the quote below:

There aren't enough [RAC] workers to provide emotional and practical and physical support that is often needed in the aged care facilities… [RAC] workers are putting [in] enormous amounts of emotional, psychological… and practical care. But we don't give them the time in their roles to do that, and often not the training to do that, given the increasing rates of dementia and other psychosocial conditions that come as we all age.

Key Informant 2

The physically and emotionally demanding nature of RAC work encapsulated by Theme 1 was conveyed by a worker association key informant and academic, and no others. This suggests that the effect on the health and safety of workers from the nature of OHS work may be overstated or not widely appreciated.

### 4.2. Casualization of RAC work

Key informants provided examples where casual employment and other non-regular work arrangements compromise workers' OHS in RAC. A typical scenario faced by RAC workers is described below:

[RAC reflects] a very insecure workforce. A lot of the workforce will work in more than one place. It's not uncommon... to hear [of RAC workers] working at two or three different facilities, working 60–70 h a week just to try to make ends meet because the pay is so poor.

Key Informant 1

The transience of RAC workers across multiple facilities was identified as a phenomenon spreading COVID-19 infections.

There is a lot of this part time work that is shining the light on… [the casualized nature of RAC work] because these workers are going to multiple facilities and therefore being vectors [for COVID-19].

Key Informant 3

Key informant 6 noted injury risk from job fatigue over a 24-h job cycle in the context of job insecurity. RAC workers may be disinclined to report fatigue-related injury where this is seen to reflect poorly on their work capacity. Key informant 6 stated that RAC workers were often reluctant “about reporting an injury [which] means that… [injury is typical] severely underreported”. Key informant 6 expressed concerns regarding reporting and monitoring of OHS risks, as well as job-specific training, explaining:

Certainly, from other industries we've seen that casualization and lack of training issues… are associated with higher [OHS] incidents and under reporting [of OHS incidents]. [There is] no reason to think that the aged care sector is different… If you have got an increasingly casualized workforce, you might still be providing training, but unless you are making sure that everyone has access to training and they are paid for the time that they spent training, you'll have people who miss out on… [training].

Key Informant 6

Employer and OHS key informants did not identify worker OHS issues associated with the casualized workforce. This suggests a possible lack of recognition of potential effects among these stakeholders.

### 4.3. Prioritization of OHS and resident safety

According to key informants, RAC service providers place a higher priority on resident-related safety with lower priority given to worker OHS. Typical examples provided by key informants included scenarios where meeting resident needs exposed workers to a risk of injury. The prioritization of resident safety over worker OHS and its resultant effects on workers is discussed below:

Residents' safety [is prioritized] as the absolute be-all and end-all… that focus is often taken… at the expense of staff health and safety. When you've got health services that have as part of their values [that] patient safety comes first, that… means that staff safety doesn't come first… Therefore, it must be second or third or fourth or something else, and that I think is a bit of a context for the staff around where they sit and how they're valued and what that means for them.

Key Informant 1

In the scenario depicted next, a worker risks injury through standard OHS work practices. The key informant's testimony describes how resident priorities become embedded and even though this creates a hazard, workers may be unwilling to speak up:

[In our research] we were doing surveys [with workers on culture] and… [in the] qualitative comments… [workers] were making comments like: ‘I almost feel as though I shouldn't report this incident, because it's someone with dementia and they can't control their arms. They didn't mean to hit me, but I did get hit…' And so, it was the downplaying of the health and safety issues for staff that was a real concern for examples like that.

Key Informant 6

Similar insights were shared on the lack of appropriate communication channels. The resident health condition described here is seen as a factor excusing injury risk exposure:

[The workers] don't like… [the incidents], but they are more accepting of when the patient hits them or yells at them because they know that that person's got dementia, so they don't report it, so the “higher ups” [(i.e., the managers)] don't know that that's happening.

Key Informant 4

However, such risky situations for workers may be changing according to key informants. RAC service providers have introduced new practices to reduce known injury problems. An example here is of lifting machines:

Increasingly, there has been a focus on worker health and safety… Back injuries in health and aged care have always been an issue, and there's more… of a focus around lifting machines [to] support… [lifting residents].

Key Informant 8

The compatibility of residents and the health and safety of workers were also noted by key informants. Verbatim interview comments explain:

Keeping yourself safe does not mean that your patients are not safe. In fact, quite the opposite. If you're safe it's going to be safer for your patients… [RAC service providers] are quite unaware of their OHS obligations, whether it's deliberately unaware, or maybe ignorant of them. I think a little bit from column A, a little bit from column B, depending on the provider, depending on the time …... But OHS is not something they think is their problem or their business.

Key Informant 1

Key informant representatives from the regulator, employer and employees, and researchers all discussed prioritization of resident safety over worker OHS. The employee representative and researchers suggested this to be an issue embedded in RAC worker culture. Of note, even when asked directly about this question, OHS industry associations did not respond in a form allowing representation of this theme. The absence raises the question of whether this situation is unique to RAC as opposed to other industry sectors.

### 4.4. Female, CALD, and aging workforce

Key informants recognized that characteristic RAC worker profiles (e.g. female, CALD, older workforce, and low paid) underpin OHS concerns. Key informants identified OHS challenges raised within RAC pertaining to the employee profile. Key informant 2 summarized this in terms of worker rights:

I would say it's [the RAC sector] a caring industry, 90% of the people in the industry are women, low paid. I know it's these three things together [that] often brings a lack of attention on worker rights, worker safety, worker conditions, etc... its [RAC OHS] traditionally been an area that's been overlooked.

Key Informant 2

CALD workers may lack assertiveness which may then be taken advantage of by RAC service providers:

There's… a significant proportion [of the RAC workforce] who are from a CALD background, who fear authorities who don't know what their rights are. That seems to suit aged care in many respects because… [CALD workers] don't ask a lot of questions. They don't put their head up - they do as they are told.

Key Informant 1

A lack of understanding of worker backgrounds and characteristics may cause a disjoint between workers and managers. This is explained in the following quote:

[Management have] a lack of understanding of the people that work for them. Aged care… has a lot of CALD - cultural, [and] linguistically diverse individuals… The work is done by women, lower education than the men… [the men] are the ones who manage the work that's done and because of that there's issues… [between RAC workers and] the white Anglo Saxon protestant males that typically run or are the CEOs of the organizations.

Key Informant 4

The aging RAC workforce was also raised as an important issue. Concern surrounded older employees' (noted below at 70 plus years) reduced physical capabilities when engaged in RAC:

I've seen carers… [who are] over 70 [years of age] and that definitely concerns me from a health and occupational health and safety perspective. I think they're more risk. I don't want to sound ageist, but I do get some concerns when I see people of that age… [in] what can be a very heavy, heavy role.

Key Informant 8

Most key informant group representatives, except industry representatives, noted safety implications due to the RAC worker profile. This lack of input from the OHS industry associations suggests that this issue may be unique to the RAC sector.

### 4.5. OHS role construction and importance

Theme 5 relays the influence of the relative position of OHS within the organization in terms of role construction and importance. Four of five key informant groups were represented in this theme. The influence of OHS in RAC was recognized by reporting lines, level of authority, and subsequent organizational power. For example, a comparison of typical OHS positions in RAC facilities with OHS positions in safety-critical work organizations is described:

The problem overall with aged care, comparative to other industries, is that it suddenly became, in terms of [COVID-19] infection, a high-risk industry. So when we look at high risk industries in the context of health and safety we see within the organizational hierarchy health and safety executives reporting to CEOs and boards; [people] who have power in the company, and who engage with heads of plants and line managers and supervisors, and have teams of health and safety people out and about… and the concept of health and safety is understood at board level… Aged care does not look at it that way at all.

Key Informant 5

The qualifications of OHS roles in RAC are also noted as being low relative to traditional high-risk sectors. OHS responsibilities in RAC are covered by less qualified individuals in lower-level roles:

[Typically], you have… [OHS positions] as second tier within the health service, which means that when you're recruiting, it's not that executive position that you might get in other industries… It means that it's very difficult to attract good people to do the health and safety role. Then it means that you don't necessarily have the best people in [the] health and safety role, which therefore perpetuates the cycle because they're not actually pushing the fact that… [OHS] needs to have a higher priority and that more needs to be done.

Key Informant 1

Also, the relative positioning of OHS in RAC compared to the positioning of comparable roles in high-risk industries is elaborated upon:

In something like mining, you find a lot of health and safety managers who have degrees in health and safety. They're very skilled at presenting to their board and getting safety as a high priority… They also tend to have more male occupational safety managers. In aged care, I came across more female safety managers and they hadn't had the training or the experience necessarily to be able to push the case for prioritization of health and safety… If… [OHS professionals] are not senior enough in the structure, it can be really hard for them to get the resources that they need [for OHS], and it's frustrating for them.

Key Informant 6

Many key informants noted distinctions between RAC OHS roles and other high-risk sectors. RAC OHS was positioned low in the organizational hierarchy and typically requires no formal OHS qualifications. A subsequent lack of power held in these positions may be reinforced through gender stereotyping. Interestingly, when asked about the positioning of OHS, the employer association key informant provided no response, suggesting a view toward obfuscation or irrelevance.

### 4.6. Tension between clinical standards and OHS approaches

Theme 6 indicated that a traditional RAC safety approach in meeting clinical standards proves suboptimal when compared with the OHS proactive approach. For example, in responding to COVID-19, RAC service providers complied with clinical standards for infection control based on currently known diseases. Employed safety practices failed to control infection spread in RAC facilities. In contrast, OHS professionals follow a ‘risk-based' proactive analysis to identify and counter new risks without necessarily relying on safety protocols embedded in standards.

In RAC, safety may be addressed at some level, based on the regulations, however, forward-looking health management was placed as a secondary priority. This is explained below:

There's a tendency for many health and safety people in aged care to do safety, but less so health. And the health supposedly sits with the clinical standards staff, but the clinical standards staff don't do the health and safety part of health.

Key Informant 5

This informant went on to explain that RAC service providers focus on compliance with clinical standards for health yet fail to consider responsibilities for worker health within OHS. The key informant also identified challenges that COVID-19 infection control presented for RAC service providers relying on clinical standards, suggesting that if qualified OHS practitioners were in place, then issues associated with infection spread through ventilation may have been addressed earlier:

What the health and safety person does, in that situation, is applies precautionary basic risk management, [and] says, ‘there are things here we don't know. We must upgrade our controls in a precautionary way. We go harder because we're not certain what's causing the problem.…. We don't keep our standards in our controls lower in the presence of increasing infections [as was the case during COVID] because that's the best evidence … that what you've got doesn't work'.

Key Informant 5

Hence, a different approach to safety and risk is applied by qualified OHS professionals when compared to the risk approach employed by clinical aged care workers. This is described:

So traditionally, infection, prevention and control is treated as a clinical matter. And it's usually done on the basis of, “is there evidence that this is a risk?” From the OHS side we are saying – “well, … is there evidence that it's not a risk?” Because if there's no evidence that it's not a risk, then we treat it as a risk until we get that evidence… That [is the] precautionary principle.

Key Informant 1

Contributions to Theme 6 were restricted to the worker association and OHS industry associations. Key Informants here conveyed tension between clinical standards and OHS approaches (Table 3). Differing approaches to safety management, these being essentially either reactive or proactive, will in most circumstances have low consequences for safety. However, when new risks emerge, or safety management is compounded by other extant factors, a proactive approach may prove preferable.

## 5. Discussion

The results from our study highlighted six main themes representing key issues for the health and safety of workers in RAC. These included: (i) the relatively hard physical and emotional nature of RAC work, (ii) a casualized RAC workforce contributing to worker vulnerability, (iii) prioritization of resident safety over employee OHS in RAC, (iv) implications for RAC arising from a predominantly female, CALD background and an aging workforce, (v) non-optimal OHS Role constructions in RAC, and vi) how the choice between clinical standards and OHS approaches may reduce RAC safety. Some themes emerged through deductive analysis and confirmed existing knowledge, such as the physical and emotional nature of RAC work, and have been identified previously (6, 8, 58, 59). However, the benefit of our hybrid approach, which also enabled inductive analysis, meant this study makes an important contribution by way of expanding understanding of existing knowledge, whereby key informants highlighted contextual factors exacerbating previously identified challenges. For example, key informants noted unsuitable workers may be employed due to a limited candidate pool. RAC workers also face resource constraints and staff cut-backs due to cost-saving initiatives which in turn exacerbate workloads and reduce available support. Workers being employed across multiple facilities was also identified as raising the potential for COVID-19 spread. Prioritization of resident safety over the health and safety of workers was also evident in the findings, with examples of resident-induced incidents that employees accepted as part of the job rather than a risk to be ameliorated. COVID-19 responses also showed clinical/care approaches as dominant over the proactive OHS approach when managing risk.

In addition, three novel and important contributions emerged through this study. First, RAC workers' health and safety have been a low priority and may continue to be unless a systematic change is pursued across the sector. The ACQS do not specify the health and safety of the workers, and the interviews in this study demonstrate this lack of priority trickles down through management decision-making and resource allocation, and also to the coal-face workers who in practice also prioritize resident health and safety over their own. Rather than RAC demonstrating a holistic and integrated model for worker and resident health and safety, it appears notions of quality, accreditation, and compliance manifest a dichotomy of resident vs. health and safety of the worker, with worker injury statistics indicating workers bear the brunt of these conditions. RAC residents certainly deserve their health and safety to be a priority, but at the moment this comes seemingly at the cost of commitment to the health and safety of workers. Resident and worker safety considerations should not be considered a trade-off situation in RAC. This study provides an evidence base with insights from representatives across a number of stakeholder groups that this dichotomy exists, it is detrimental to the health and safety of workers, and we argue this issue is worthy of further research.

Second, despite recently being escalated to a safety-critical status (largely due to COVID infections) the extant governance of the health and safety of the RAC workers is yet to reflect equivalent approaches to that of other safety-critical sectors such as aviation, oil and gas production or nuclear power generation. Previous research has highlighted the importance of having an influence on senior leaders as well as workers' health and safety considerations in strategic or funding decisions (39, 40, 60). However, our findings highlight that RAC OHS roles typically lack suitable qualifications and seniority to influence decision-making and inform the step-change required for the health and safety of the RAC worker to curb workplace injuries and create safer environments. These inadequacies of RAC OHS governance are complicated by the context of RAC worker health and safety, for instance, that the RAC workforce is at risk of injury. Our study confirms prior knowledge that the nature of work, the workforce characteristics, and the casualization of the workforce are challenges, however, combined with governance deficiencies, these issues create a “perfect storm” for workplace injuries. Our study indicates that unless something is done to address these challenges, workplace injuries of RAC workers are likely to get worse before we see improvement.

Third, our study identified preferred modes of operation in RAC workers' health and safety whereby traditional dominant health sector-related logic and clinical methods were championed over a proactive worker health and safety approach, and the deficit of such approaches was highlighted with the mismanagement of COVID infections by RAC service providers (48, 49). While infection control is a specific requirement within the ACQS (17, 28), RAC service providers largely applied their typical clinical methods in responding to COVID infections. However, COVID-19 presented a new and unprecedented hazard where standard approaches proved sub-optimal. A proactive approach using OHS logic may have been preferable in using expert knowledge to translate from general evidence on virus spread to improving ventilation within RAC facilities (61). Our study highlighted that reliance on traditional dominant clinical methods, combined with a lack of worker health and safety governance, low prioritization of the health and safety of workers, and a vulnerable workforce with limited ability to speak up about broken systems, was ill-prepared for an external force such as COVID-19. Unless significant change occurs in the sector, they may well be ill-prepared for subsequent challenges.

## 6. Limitations

Study themes were limited to data provided by selected key informants and their available knowledge. Unknown bias may have been introduced by key informants' responses influenced by social desirability or adherence to their professional positions. Theme development was based on subjective interpretations by the researchers, though direct quotes do provide objective supporting evidence. While the semi-structured nature of the interviews allowed for key informants to provide their own views and perspectives, further studies may use the results reported here as a guide to analyzing RAC holistically using a systems perspective. Future related studies may also focus on methods for installing OHS institutional logic throughout RAC. While this study did not seek to explore the influence of key informants' gender or age on their insights/responses, there may also be potential for future studies to explore how socio-cultural factors impact reported insights and/or experiences of the health and safety of RAC workers.

## 7. Practical implications

This study contributes the much-needed knowledge of the complex nature and interactions of RAC and worker health and safety from the perspective of a range of key informants and provides practical implications and suggestions for future research. Practical implications are evident at the policy, process, and practice levels. For instance, the absence of specific guidelines related to worker health and safety in the ACQS is an oversight that appears to enable systematic undervaluing and de-prioritization of worker health and safety and needs to be addressed by government and relevant sector stakeholders. In terms of process, a key initiative would be incentivizing the implementation of appropriate worker health and safety governance structures for the sector, and by RAC service providers. In terms of practice, a key initiative would be employee groups and worker unions engaging in a campaign to inform RAC workers of their rights at work and practical solutions to respond to workplace demands in ways that simultaneously uphold resident and worker health and safety.

In addition, the results and findings present several relevant pathways for future research. There is an opportunity to study the impacts of the RAC funding model and its association and prioritization within the quality standards. There is also an opportunity to better understand RAC leadership and its approach to managing conflicting priorities within the complexity of RAC workplaces. Further research into these significant challenges may help stakeholders and policymakers within the sector better understand how to integrate worker health and safety to improve resident care.

## 8. Conclusion

Worker health and safety in RAC is complex and influenced by a range of themes, each of which can impact worker health and safety performance within a RAC facility. Of particular importance is how worker health and safety are prioritized, based on how the sector is funded and accredited. This arrangement can influence the governance of worker health and safety and the institutional logic that guides decisions about worker health and safety. Without appropriate governance structures with OHS representatives in positions of influence with senior decision-makers, worker health and safety will be less effective in demonstrating the value of their professional logic across the organization. Further research investigating the prioritization of worker health and safety and residents is best addressed and the influence of leadership in the sector will help in understanding how to incorporate and integrate worker health and safety into RAC sector frameworks. This will also aid in encouraging RAC service providers to better understand the importance of worker health and safety and its positive impact on organizational decisions.

## Data availability statement

The datasets presented in this article are not readily available because interview data only. Requests to access the datasets should be directed to LS, e.seaward@federation.edu.au.

## Ethics statement

The studies involving human participants were reviewed and approved by the Federation University Human Research Ethics Committee. The patients/participants provided their written informed consent to participate in this study.

## Author contributions

LS, DM, and AT contributed to conception and design of the study. LS identified experts, identified interview topics, conducted and analyzed all interviews, and wrote the first and subsequent drafts of the manuscript. DM and AT contributed to interview topics and review of themes. All authors contributed to manuscript revision, read, and approved the submitted version.
